# Expert consensus on pulpotomy in the management of mature permanent teeth with pulpitis

**DOI:** 10.1038/s41368-024-00333-9

**Published:** 2025-01-07

**Authors:** Lu Zhang, Chen Lin, Zhuo Chen, Lin Yue, Qing Yu, Benxiang Hou, Junqi Ling, Jingping Liang, Xi Wei, Wenxia Chen, Lihong Qiu, Jiyao Li, Yumei Niu, Zhengmei Lin, Lei Cheng, Wenxi He, Xiaoyan Wang, Dingming Huang, Zhengwei Huang, Weidong Niu, Qi Zhang, Chen Zhang, Deqin Yang, Jinhua Yu, Jin Zhao, Yihuai Pan, Jingzhi Ma, Shuli Deng, Xiaoli Xie, Xiuping Meng, Jian Yang, Xuedong Zhou, Zhi Chen

**Affiliations:** 1 Department of Cariology and Endodontics, Wuhan University & State Key Laboratory of Oral & Maxillofacial Reconstruction and Regeneration, Key Laboratory of Oral Biomedicine Ministry of Education, Hubei Key Laboratory of Stomatology, School & Hospital of Stomatology, Wuhan University, Wuhan, China; 2 Department of Endodontics, Stomatological Hospital of Xiamen Medical College, Xiamen, China; 3 Stomatology Hospital, School of Stomatology, Zhejiang University School of Medicine, Hangzhou, China; 4 Department of Cariology and Endodontology, Peking University School and Hospital of Stomatology, National Center of Stomatology, National Clinical Research Center for Oral Diseases & National Engineering Laboratory for Digital and Material Technology of Stomatology, Beijing Key Laboratory of Digital Stomatology & Research Center of Engineering and Technology for Computerized Dentistry Ministry of Health & NMPA Key Laboratory for Dental Materials, Beijing, China; 5 State Key Laboratory of Oral & Maxillofacial Reconstruction and Regeneration, National Clinical Research Center for Oral Diseases, Shaanxi Key Laboratory of Oral Diseases, Department of Operative Dentistry & Endodontics, School of Stomatology, The Fourth Military Medical University, Xián, China; 6 Center for Microscope Enhanced Dentistry, Beijing Stomatological Hospital, Capital Medical University, Beijing, China; 7 Department of Operative Dentistry and Endodontics, Hospital of Stomatology, Guanghua School of Stomatology, Guangdong Provincial Key Laboratory of Stomatology, Sun Yat-Sen University, Guangzhou, China; 8 Department of Endodontics, Shanghai Ninth People’s Hospital, Shanghai Jiao Tong University School of Medicine; College of Stomatology, National Clinical Research Center for Oral Diseases, National Center for Stomatology, Shanghai Key Laboratory of Stomatology, Shanghai Jiao Tong University, Shanghai, China; 9 College & Hospital of Stomatology, Guangxi Medical University, Nanning, China; 10 Department of Endodontics, School of Stomatology, China Medical University, Shenyang, China; 11 State Key Laboratory of Oral Diseases & National Center for Stomatology & National Clinical Research Center for Oral Diseases & West China Hospital of Stomatology, Sichuan University, Chengdu, China; 12 School of Stomatology, The First Affiliated Hospital of Harbin Medical University, Harbin Medical University, Harbin, China; 13 Department of Stomatology, Air Force Medical Center, The Air Force Medical University, Beijing, China; 14 School of Stomatology, Dalian Medical University, Dalian, China; 15 Department of Endodontics, Stomatological Hospital and Dental School of Tongji University, Shanghai Engineering Research Center of Tooth Restoration and Regeneration, Shanghai, China; 16 Department of Endodontics, Beijing Stomatological Hospital, School of Stomatology, Capital Medical University, Beijing, China; 17 Department of Conservative Dentistry and Endodontics, Shanghai Stomatological Hospital, School of Stomatology, Shanghai Key Laboratory of Craniomaxillofacial Development and Diseases, Fudan University, Shanghai, China; 18 Department of Endodontics, Institute of Stomatology, Affiliated Hospital of Stomatology, Nanjing Medical University, Nanjing, China; 19 Department of Endodontics, The First Affiliated Hospital of Xinjiang Medical University, Urumqi, China; 20 Department of Endodontics, School and Hospital of Stomatology, Wenzhou Medical University, Wenzhou, China; 21 Department of Stomatology, Tongji Hospital, Tongji Medical College, Huazhong University of Science and Technology, Wuhan, China; 22 Department of Endodontology, Hunan Xiangya Stomatological Hospital, Central South University, Changsha, China; 23 Department of Endodontics, School and Hospital of Stomatology, Jilin University, Changchun, China; 24 Department of Endodontics, The Affiliated Stomatological Hospital of Nanchang University, Nanchang, China

**Keywords:** Pulpitis, Endodontics

## Abstract

Pulpotomy, which belongs to vital pulp therapy, has become a strategy for managing pulpitis in recent decades. This minimally invasive treatment reflects the recognition of preserving healthy dental pulp and optimizing long-term patient-centered outcomes. Pulpotomy is categorized into partial pulpotomy (PP), the removal of a partial segment of the coronal pulp tissue, and full pulpotomy (FP), the removal of whole coronal pulp, which is followed by applying the biomaterials onto the remaining pulp tissue and ultimately restoring the tooth. Procedural decisions for the amount of pulp tissue removal or retention depend on the diagnostic of pulp vitality, the overall treatment plan, the patient’s general health status, and pulp inflammation reassessment during operation. This statement represents the consensus of an expert committee convened by the Society of Cariology and Endodontics, Chinese Stomatological Association. It addresses the current evidence to support the application of pulpotomy as a potential alternative to root canal treatment (RCT) on mature permanent teeth with pulpitis from a biological basis, the development of capping biomaterial, and the diagnostic considerations to evidence-based medicine. This expert statement intends to provide a clinical protocol of pulpotomy, which facilitates practitioners in choosing the optimal procedure and increasing their confidence in this rapidly evolving field.

## Introduction

Pulpitis is the pulp inflammation due to infection or injury.^1^ The clinical symptoms of pulpitis generally manifest with pain, especially to hot and cold stimuli, or exacerbated by lying down.^2^ According to a survey by the Chinese local report,^3^ the incidence of pulpitis is approximately 30%, which imposes a substantial economic burden on individuals, medical institutions, and the government.

The treatment approaches for pulpitis went through a series of developments, which can be traced back to extracting the affected tooth or attempting to alleviate pain via various remedies (such as applying acids or alkalies to burn the dental nerves) among Egyptians and Greeks. With the development of endodontology, the treatment strategies for infected dental pulp involve two main approaches.^4^ One way is the “thorough removal of the infection inside the root canal”, represented by root canal treatment (RCT), which came out early in the mid-19th century.^5^ Another way is “rendering the pulp tissues free of infection”, which was initially represented by mummification in the late 19th century. Subsequently, in the mid-20th century, Wang et al.^6^ proposed the resinifying therapy (RT) with Chinese characteristics.^7,8^ Nevertheless, due to the comparatively poor long-term outcomes relative to RCT, both therapies have gradually been relegated to historical footnotes, with RCT emerging as the prevailing treatment for pulpitis.^9–11^ However, RCT also has some drawbacks, such as high costs, long treatment duration, prone to root fracture, and, most importantly, the removal of all dental pulp tissue.

**Fig. 1:**
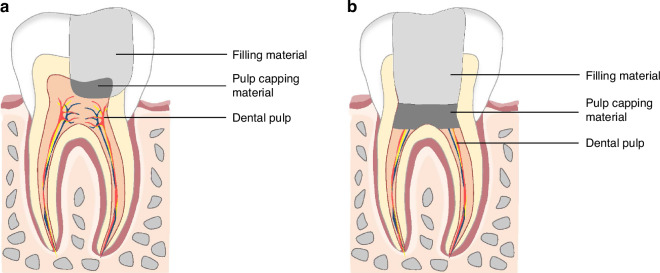
Pulpotomy divides into PP (**a**) and FP (**b**)

Vital pulp therapy (VPT) is a conservative approach that preserves the healthy pulp following injury caused by trauma, caries, or restorative procedures.^12–14^ The objective of VPT is to stimulate reparative dentin formation and maintain pulp vitality, thereby preserving the normal physiological function of the tooth.^15–17^ The VPT procedure encompasses pulp capping and pulpotomy. Pulp capping is generally applied in treating deep caries, while pulpotomy is primarily used for treating pulpitis confined to the coronal pulp. Pulpotomy is further categorized as partial and full pulpotomy (Fig. 1).^18,19^

- Partial pulpotomy (PP)
  PP is defined as removing a small portion of coronal pulp tissue after exposure, followed by applying a biomaterial directly onto the remaining pulp tissue before placement of a permanent restoration.^18,20^
- Full pulpotomy (FP)

FP is defined as the complete removal of the coronal pulp and application of a biomaterial directly onto the pulp tissue at the level of the root canal orifice(s) prior to placement of a permanent restoration.^18,20^

Cvek first reported the treatment of a traumatically exposed young permanent tooth using PP in 1978.^21^ In 1993, Cvek et al.^22^ reported on the application of PP in young permanent teeth with deep carious for the first time. Pulpotomy in immature permanent teeth could achieve a success rate comparable to apexification. Recent studies increasingly support pulpotomy as a potential alternative to RCT, even for mature permanent teeth with irreversible pulpitis.^19,23–25^

Nowadays, both the European Society of Endodontology (ESE) and the American Association of Endodontists (AAE) have issued a position statement on VPT.^18,20^ The shift in focus from aggressive pulp removal to minimally invasive approaches reflects the recognition of the importance of preserving natural tooth structure and optimizing long-term patient-centered outcomes.

This statement represents the consensus of an expert committee convened by the Society of Cariology and Endodontics, Chinese Stomatological Association. This statement intends to address the current evidence to support the application of pulpotomy on mature permanent teeth with pulpitis from a biological basis, the capping biomaterials, and the diagnostic considerations to evidence-based medicine. Besides, it aims to provide the practitioner with relevant clinical guidance in this rapidly evolving field.

## Defense and repair potential of dental pulp complex

**Fig. 2:**
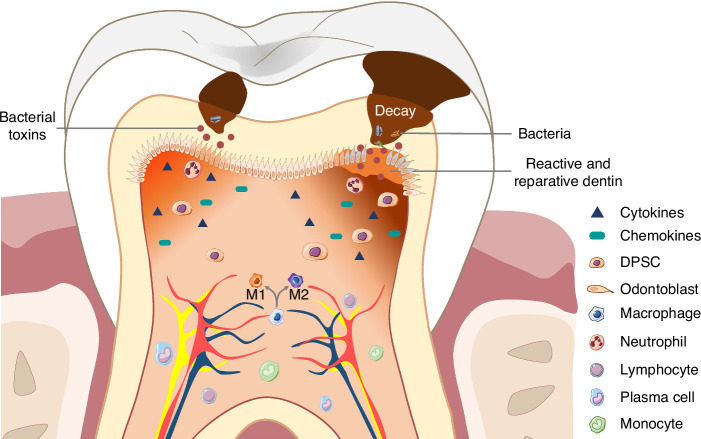
Biological mechanism for dental pulp complex against inflammation

Conventional viewpoints suggest that the low compliance and deficient collateral circulation of the pulp chamber prevent the pulp from tolerating increased pressure during pulp inflammation and limit its ability to deliver immune components to the injury site.^26–29^ However, dental pulp demonstrates adaptability to moderate pressure increments, and a robust immune defense response is concurrently activated during inflammation.^30–32^ Upon penetration of virulence factors through the dentinal tubules, odontoblasts discern and instigate the intrinsic immune defense response.^33–35^ With the progression of pulpal inflammation, the release of a substantial array of inflammatory mediators and cytokines, orchestrating an enhanced recruitment of immune cells, including neutrophils, macrophages, lymphocytes, plasma cells, and monocytes, towards the epicenter of inflammation.^36–38^ Concurrently, dental pulp stem cells (DPSCs) are mobilized to the inflammatory site, where they undergo differentiation into odontoblast-like cells. Reparative dentin formed by odontoblast-like cells and reactive dentin secreted by odontoblasts form a dentin bridge to block bacterial invasion (Fig. 2).^39,40^ The removal of the infected crown pulp and the formation of the dentin bridge could provide a protective barrier for the remaining vital pulp.^41^

## Capping biomaterials

Common clinical pulp capping materials include calcium hydroxide (CH), mineral trioxide aggregate (MTA), iRoot, and Biodentine. CH is no longer the preferred pulp capping material due to the formation of incomplete dentin bridges, poor adhesion, and high solubility.^42–44^ MTA demonstrates a superior success rate (around 85%) and elicits fewer pulpal inflammatory reactions than CH (around 71%).^45^ MTA has been shown to possess good sealing, biocompatibility, antimicrobial properties and induce hydroxyapatite formation.^46,47^ Biodentine, in comparison to MTA, exhibits analogous biological properties.^48–50^ A meta-analysis study encompassing a follow-up duration of 2–3 years revealed that Biodentine achieved a success rate of 86% in the treatment of mature permanent teeth with cariously exposed pulp, higher than MTA (84%) and CH (59%).^51^ Besides, the novel nanobioceramic material, iRoot, is recognized for its commendable biocompatibility, antimicrobial properties, flowability, and hydrophilicity.^52–55^ A retrospective study indicated that the success rate of iRoot BP Plus for pulpotomy could be up to 99% in 12–24 follow-up periods.^56^

Evidence-based medicine robustly substantiates the clinical value of calcium silicate cement (MTA, Biodentine, and iRoot) as pulp-capping materials in pulpotomy. However, considering the discoloration property of MTA, it is recommended in non-esthetic zones. Conversely, iRoot and Biodentin are suitable choices for use in the anterior aesthetic zone. Besides, the rapid development of domestically produced materials in China with excellent performance and affordable prices, is expected to to meet the significant demand in clinical practice.

## The classification of pulpitis

The conventional categorization of pulpitis is mainly based on clinical manifestations according to the AAE standard.^20^ “Reversible pulpitis” is defined as the localized inflammatory response to caries approximating the pulp space, suggesting an absence of bacterial invasion within the pulp, and displaying discomfort upon exposure to cold or hot stimuli but without spontaneous pain. In contrast, “irreversible pulpitis” is characterized by spontaneous pain triggered by bacterial-related stimuli, showing lingering pain after a stimulus.^57^ However, histological analyses of the pulpitis continuum reveal an indistinct threshold between reversible and irreversible states.^58^ Thus, pulpitis can be interpreted as a disease of temporal and spatial grading, where inflammation spreads to the crown pulp first while the root pulp remains healthy.

Wolters proposed a novel diagnosis terminology for pulpitis, which categorized pulpitis as “initial”, “mild”, “moderate”, and “severe” stages with corresponding treatment strategies (Table 1).^59^ This new system by Wolters moves beyond the simplistic binary categorization of pulpitis as either “reversible” or “irreversible”. Instead, it comprehensively considers the continuous stages of pulpitis accompanied by different severity of inflammation.

**Table 1:** Diagnostic classification of pulpitis between AAE^20^ and Wolters et al.^59^

AAE classification	Wolters classification	Clinical symptoms	Histological situation	Therapy
Reversible pulpitis	Initial pulpitis	Heightened but not lengthened response to the cold test. No spontaneous pain or percussion sensitivity.	Limited local inflammation is confined to a coronal pulp.	Indirect pulp capping
Reversible pulpitis	Mild pulpitis	Heightened and lengthened reaction to cold, warm, and sweet stimuli that can last up to 20 s. No spontaneous pain but possible percussion sensitivity.	Limited local inflammation is confined to a coronal pulp.	Indirect pulp capping
Irreversible pulpitis	Moderate pulpitis	Strong, heightened, and prolonged reaction to cold, which can last for minutes. Spontaneous dull pain that can be suppressed with pain medication, and possible percussion sensitivity.	Extensive local inflammation confined to coronal pulp.	PP/FP
Irreversible pulpitis	Severe pulpitis	Clear pain reaction to warmth and cold stimuli. Severe spontaneous sharp or dull pain and very sensitive to percussion.	Extensive local inflammation of coronal pulp possibly extending into root canals.	FP-if hemostasis can be achieved. RCT-if hemostasis failed.

Compared to the AAE classification, the new Wolters classification is guiding clinicians in choosing more conservative therapeutic options. A prospective clinical study also demonstrated that the novel Wolters’ classification was more conducive to diagnosing and treating pulpitis than the traditional AAE classification.^60^ However, insufficient objective evidence exists to determine the superiority of the two classification systems for pulpitis. Besides, the new Wolters classification is more complex, presenting challenges for clinical implementation and raising questions about its suitability in China. Thus, there are no recommendations for the classification of pulpitis in China currently.

## Treatment options for pulpitis

### Pulpotomy or RCT

Drawing upon extensive clinical evidence, multiple systematic reviews and meta-analyses have consistently demonstrated that pulpotomy yields clinical and radiographic success rates comparable to those of RCT, supporting a reliable option for pulpotomy even in mature permanent teeth with irreversible pulpitis (Table 2).^61–72^ RCT is determined according to the established protocol,^73^ while pulpotomy may change the plan during the operation, increasing intermediate decision-making. Besides, pulp inflammation, reassessed by bleeding control and clinical tissue appearance during operation, is significant for clinicians choosing pulpotomy or RCT.

**Table 2:** Systematic reviews and meta-analysis on pulpotomy in mature permanent teeth

Author	Year	Diagnosis	Treatment method	Included studies	Conclusions
Alqaderi et al.^61^	2016	Carious exposed permanent posterior teeth	FP	6	FP had a favorable success rate in treating various vital pulp exposures of permanent mature teeth with closed root apices.
Li et al.^62^	2019	Carious exposed pulps; irreversible pulpitis	FP	26	Pulpotomy is a prospective substitute for RCT in managing permanent teeth with carious pulp exposures, even in permanent teeth with irreversible pulpitis.
Cushley et al.^63^	2019	Irreversible pulpitis	FP	8	High success for pulpotomy for teeth with signs and symptoms of irreversible pulpitis.
Elmsmari et al.^64^	2019	Carious exposed permanent posterior teeth	PP	11	A PP has high success rates in treating carious exposed permanent posterior teeth for up to 2 years.
Zafar et al.^65^	2020	Irreversible pulpitis	Pulpotomy/RCT	6	Pulpotomy can be an alternative option for mature permanent teeth with irreversible pulpitis.
Lin et al.^66^	2021	Carious exposed permanent posterior teeth	FP/PP	11	FP and PP demonstrated a high success rate in treating carious exposed vital pulp of mature permanent molars.
Leong et al.^67^	2021	Carious exposed permanent teeth	DPC/FP	6	VPT is a reliable treatment option for permanent teeth with carious pulp exposure.
Santos et al.^68^	2021	Irreversible pulpitis	FP/PP	12	VPT for irreversible pulpitis has favorable outcomes, with success rates ranging from 81% to 90%.
Albaiti et al.^69^	2022	Carious exposed permanent posterior teeth	PP	4	PP showed a high success rate in treating carious exposed permanent posterior teeth for up to 24 months.
Ather et al.^70^	2022	Irreversible pulpitis	Pulpotomy	11	The first meta-analytical study that determines the clinical outcome of pulpotomy for carious teeth with irreversible pulpitis and its predictors for success.
Skitioui et al.^71^	2023	Irreversible pulpitis	FP	4	FP appears to have a high success rate as a permanent treatment of irreversible pulpitis and could be considered an alternative to RCT.
Tomson et al.^72^	2023	Permanent teeth with pulpitis characterized by spontaneous pain	Pulpotomy/RCT	2	Pulpotomy is a definitive treatment modality that is as effective as RCT.

*DPC* direct pulp capping, *PP* partial pulpotomy, *FP* full pulpotomy, *RCT* root canal therapy, *VPT* vital pulp therapy

### PP or FP

The difference between PP and FP is the amount of pulp tissue removal depends on the boundary and degree of pulp inflammation. Nevertheless, precise diagnostic methods for pulp vitality are currently lacking. The current pulp vitality test detects the physiological function of the sensory nerve to cold, heat, or current rather than the pulp’s vitality (blood circulation) and inflammatory state. Recently, a systematic review and meta-analysis of the diagnostic accuracy of dental pulp tests have shown that temperature tests and electric pulp testing alone are not very accurate in determining pulp vitality, even if cold tests are more sensitive than heat tests.^74^ Laser Doppler flowmetry (LDF) and pulse oximeter (PO) are the most accurate methods, but promotion has some difficulties.^75^

Based on current clinical experience, it is recommended that the combined electric pulp and cold tests applied in pre-operation^57^ and the hemorrhage control and clinical tissue appearance with magnification during operation to guide clinicians in assessing pulp inflammation and choosing the optimal procedures.

The tooth has normal pulp vitality or is diagnosed with reversible pulpitis, there is no statistically significant difference in the prognosis between PP and FP,^76–78^ making PP a minimally invasive choice. For mature teeth with pulp exposure, there is no direct evidence that PP is more suitable than FP. However, FP demonstrates better and more predictable clinical outcomes in cases of irreversible pulpitis. A meta-analysis showed that the clinical and radiographic success rates of FP are higher than those of PP, ranging from 92.2% to 99.4% and 78.2% to 80.6%, respectively.^66^ Table 3 further compares the Pros and Cons of PP and FP.

**Table 3:** Pros and cons of PP and FP

Procedure	Pros	Cons
PP	Preserving partial coronal pulp benefits electric pulp testing and aligns with minimally invasive treatment.	1. Incomplete eradication of infection may hinder subsequent tissue healing. 2. The technique of PP is more sensitive than FP.
FP	1. Thoroughly remove inflammation and infection, which facilitates better tissue healing. 2. The pulpal chamber floor at the root canal orifice provides a solid foundation for placing pulp capping materials. 3. Easier to perform compared with PP.	1. Complete removal of the coronal pulp prevents the possibility of conducting electric pulp testing. 2. Hard tissue deposition may cause root canal calcification in the long-term follow-up, making subsequent RCT more challenging if the initial treatment fails.

## Clinical procedure of pulpotomy

**Fig. 3:**
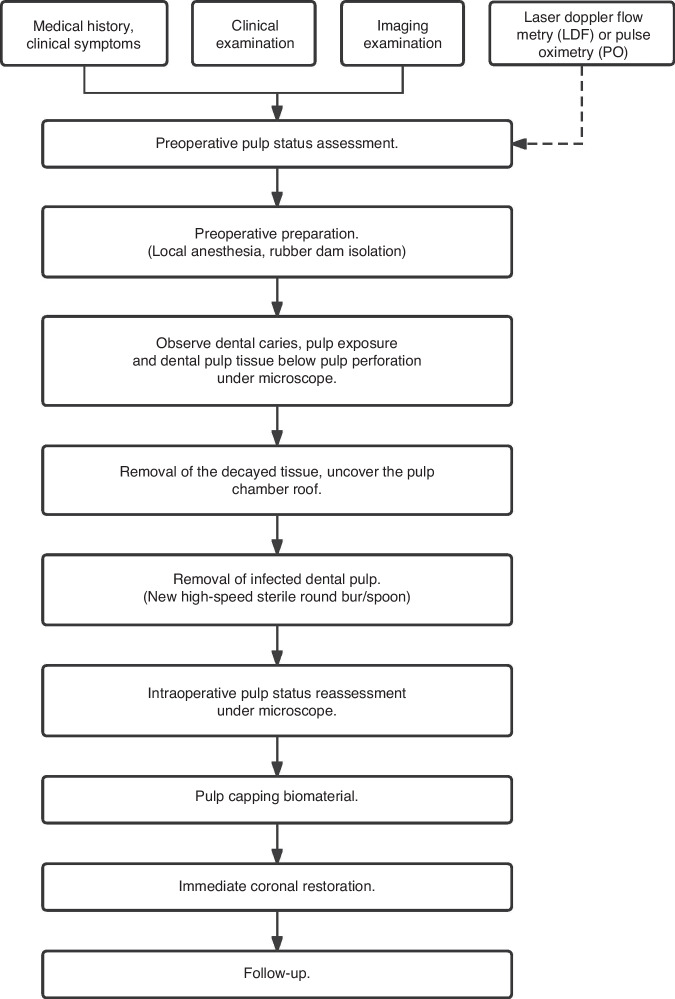
Clinical procedure of pulpotomy

To optimize therapeutic outcomes, the clinical procedure of pulpotomy can be referred to as follows (Fig. 3).

- Preoperative pulp status is evaluated based on the patient’s medical history, clinical manifestations, physical findings, and radiographic information, and when necessary, combined with the pulp blood supply status monitored by LDF or PO.^74,79^
- The carious dentine tissue is removed following local anesthesia and rubber dam isolation.
- The infected pulp tissue is removed using a new high-speed bur or a minimally invasive bur under a microscope, continuously cooling with sterile water or saline.
- Direct observation of the bleeding surface and volume under the microscope during operation could help to reassess the pulp status and determine the optimal procedures.
- Hemostasis with 1.5%–5% sodium hypochlorite (NaClO) solution is recommended.^80,81^ The time of hemostasis should be controlled within 5–10 min.^81,82^
- Bioceramic materials such as MTA, iRoot BP Plus, or Biodentine with a more than 1.5 mm thickness are immediately covered on the exposed pulp tissue^20,48,77,78,81–90^ after successful hemostasis.
- Immediate restoration is recommended,^20^ which employs a 2 mm glass ionomer cement (GIC) with composite resin covered on top.^91^

Among the procedures, microscopic operation, aseptic protocol (including rubber dam isolation, replacing the bur for pulp removal, and disinfection irrigants), hydrophilic bioactive pulp capping materials, and the sound immediate restoration are critical to the prognosis of pulpotomy.^18^ Caries risk factors may affect the prognosis of pulpotomy, and more relevant evidence is needed to support this conclusion in the future, but it is lacking at present.

## Clinical outcome assessment

The evaluation of pulpotomy efficacy in mature permanent teeth is divided into clinical and radiographic criteria involving examination of the symptoms and signs of the affected tooth, pulp electrical test, temperature test, and X-ray image at 6, 12, and 24 months and, if needed, once a year for the next four years (Table 4).^18,92–94^ LDF or PO may also be employed to monitor the blood supply of the dental pulp for a long time. Besides, from a histological perspective, successful pulp preservation therapy is characterized by developing a restorative dentin bridge. Previous studies have shown a correlation between the formation of dentin bridges with a higher success rate.^56,95,96^

**Table 4:** Evaluation criteria of pulpotomy in mature permanent teeth

Criteria	Primary outcome
Clinical criteria	No symptoms like pain, soft tissue swelling, or sinus tract. The tooth maintains vitality, and the pulp electrical test is normal under feasible conditions.
Radiographic criteria	No imaging evidence of internal or external root resorption, periapical radiolucency, or abnormal calcification. A reparative dentin bridge is formed, or if no dentin bridge formation is observed, it does not mean failure and should be recalled regularly.

## Consensus-based recommendations

This article presents a statement on pulpotomy for mature permanent teeth pulpitis:

- Pulpotomy is a potential alternative option to RCT for mature permanent teeth with pulpitis, which depends on the precise diagnosis of pulp vitality made by clinicians.
- Pulp inflammation reassessed by direct observation of hemorrhage control and clinical tissue appearance with magnification during operation is recommended to guide clinicians in choosing the optimal procedures.
- FP is recommended for cases where it is difficult to judge the scope of infected coronal pulp.
- An advanced, rigorous aseptic protocol (such as rubber dam isolation, replacing the bur for pulp removal, and disinfection irrigants) should be applied during the operation.
- Hydrophilic bioactive pulp capping materials and immediate restoration are recommended.

## Summary and expectation

Overall, this expert consensus summarizes the current evidence to support the application of pulpotomy from a biological basis to evidence-based medicine. The clinical protocol of pulpotomy facilitates practitioners in choosing the optimal procedure and increasing their confidence to apply pulpotomy in clinics. For mature permanent teeth with fully formed root apex, preserving the dental pulp maintains its immune defense mechanisms and inherent self-repair capabilities, which are crucial for the long-term viability of the tooth.

However, some limitations, areas of controversy, low-quality evidence, and uncertainties may impede the application of pulpotomy. First, there needs to be more accurate methods to judge pulp vitality. The urgent demand calls for developing low-cost, user-friendly, high-efficiency, and high-precision methods to evaluate pulp vitality. For example, pulse oximetry should be improved to increase its portability and fit in the mouth’s anterior and posterior teeth.^97^ Recently, the molecular test kit to analyze pulpitis biomarker levels may be applied as a diagnostic tool for armchair.^98^ However, the effectiveness of biomarker detection remains to be determined,^99,100^ and such an approach must establish an accurate inflammatory threshold. In addition, applying artificial intelligence to research models of pulpitis, thus using big data to determine the status of the patient’s pulp vitality, could enable personalized and integrated VPT for patients with pulpitis.^101^ Second, more accurate diagnostic methods could be applied to develop other pulpitis classifications to instruct clinics. Third, in terms of basic research, an in-depth understanding of the biological changes after pulpotomy and the effects of different pulpotomy tools on the pulp will help to understand the complications of pulpotomy and improve the pulpotomy tools to make them more standardized. Finally, the majority of existing pulpotomy research is based on short-term clinical studies or single-arm prospective studies, introducing the results at a high risk of bias. Considering the large number of pulpitis patients in China, more high-quality multi-center and large-sample clinical studies and relevant evidence-based medicine on pulpotomy are needed.
